# Social connectedness and engagement in preventive health services: an analysis of data from a prospective cohort study

**DOI:** 10.1016/S2468-2667(18)30141-5

**Published:** 2018-08-22

**Authors:** Mai Stafford, Christian von Wagner, Sarah Perman, Jayne Taylor, Diana Kuh, Jessica Sheringham

**Affiliations:** a The Health Foundation, London, UK; b MRC Unit for Lifelong Health and Ageing, University College London, London, UK; c Department of Behavioural Science and Health, University College London, London, UK; d National Institute for Health Research Collaborations for Leadership in Applied Health Research and Care North Thames, Department of Applied Health Research, University College London, London, UK; e Screening Quality Assurance Service, Public Health England, London, UK; f London Borough of Hackney & City of London Corporation, Hackney Service Centre, London, UK

## Abstract

**Background:**

Evidence of the possible health benefits of social connectedness is increasing. We aimed to examine poor social connectedness as a possible barrier to participation in preventive health services among older people (aged 53–69 years).

**Methods:**

We analysed data from a prospective cohort study of 5362 socially stratified births from the Medical Research Council National Survey of Health and Development enrolled in England, Scotland, and Wales in March 1946. At ages 68–69 years, participants reported participation in blood pressure and cholesterol measurement, eyesight and dental check-ups, influenza immunisation, and bowel and breast cancer screening. Our primary outcome measure summed participation across all these tests and services at ages 68–69 years. We tested associations between structural and functional social connectedness from ages 53 years to 69 years and total count of participation in these preventive services in Poisson models controlling for sex, education, occupational class, employment, chronic illnesses, and general practitioner consultations for health problems.

**Findings:**

940 (44%) of 2132 participants attended all preventive services within the recommended timeframes. At ages 68–69 years, being unmarried or not cohabiting (incident rate ratio [IRR] 1·33, 95% CI 1·20–1·47) and small personal social networks (IRR 1·51, 1·32–1·71) were independently associated with non-participation in more services, with associations consistent across most services. High social relationship quality at ages 68–69 years (IRR 0·91, 95% CI 0·87–0·95) and increasing social relationship quality from ages 53 years to 69 years (IRR 0·93, 0·89–0·97) were associated with low risk of non-participation.

**Interpretation:**

Individuals with poor social connectedness appear to be at greater risk of not engaging in the full range of preventive services than individuals with good social connectedness. Improvement of access to social contacts and networks in older ages is already recommended for the maintenance of good mental health. This study suggests that social connectedness could also improve participation in a wide range of preventive health services, and hence could improve use of the health-care system and population health.

**Funding:**

UK Medical Research Council.

## Introduction

Prevention and early detection of diseases is of high priority in the context of rising health-care costs. Preventive health-care programmes, including routine health checks, immunisations, and cancer screening, are publicly funded in several countries, including the UK. Participation in these programmes, termed preventive health services, is typically less than the recommended rates; therefore, research is needed to identify barriers to participation. We considered poor social connectedness as a possible barrier to participation in screening and preventive health services. Engagement in preventive health care might be one of the pathways linking social connectedness to mortality and other outcomes.^1^, ^2^

Social connectedness has structural and functional dimensions. The structural dimension covers the quantity and form of a person's relationships, whereas the functional dimension covers the quality of those relationships and the support derived. Structural and functional aspects of social connections might promote participation in preventive health services through multiple pathways, including providing informational, appraisal, and practical support that increases awareness of and facilitates access to these services, reduces anxiety related to results of screening tests, and promotes a sense of personal control.^2^ Social connectedness could reinforce a sense of meaning gained from social roles and encourage a sense of belonging that motivates self-care. Additionally, personal social networks are a context for providing social influence, including norms of help-seeking and preventive health behaviours.^3^ Large social networks and increased social contact could increase exposure to others who have participated in the preventive health service or who have had the disease and are therefore aware of susceptibility and the benefits of early detection.^4^ Protection against contagious diseases for and from friends and family might motivate participation in immunisation.^5^

Evidence suggests increased engagement among socially-connected people in preventive health services compared with those who are not socially connected. Large personal social networks,^6^ living with a spouse,^7^, ^8^, ^9^ and high perceived social support^8^ are positively associated with participation in routine health checks. Individuals who live with another person^10^ and those who have children^5^ are more likely to be immunised against influenza than those who live alone: this probability increases with the number of daily social contacts^11^ and participation in social activities.^6^ Being married or living with a partner is also associated with participation in cancer screening;^12^, ^13^ however, evidence is conflicting with some studies finding no association or an inverse association between social connectedness and engagement in preventive health services.^14^, ^15^, ^16^ Marital status has been a focus of these studies, with the functional dimension having received much less attention than has the structural dimension, and studies of social support primarily only include women.^17^, ^18^ Studies are predominantly cross-sectional, and, to our knowledge, length of exposure to or dynamics of social connectedness, which might be important in mid-to-late adulthood with changing employment and social roles, have not been explored in relation to preventive health care. Some benefits of social connectedness, such as enhanced sense of belonging and health-promoting behavioural norms, might accumulate over time; therefore, long exposure to social connectedness should be more beneficial than concurrent exposure. However, practical and informational support might need to be current to effect use of preventive services. Additionally, improvements in social connected-ness could be associated with increased engagement in preventive health services. Such an association would provide strong evidence, albeit observational, of the potential value of social connectedness interventions.

**Research in context**

**Evidence before this study**

We searched PubMed, PsychINFO, and Social Science Citation Index from database conception until Oct 18, 2017, for articles on social connectedness with the search terms “social relationship”, “social support”, “social network”, “social contact”, “marital”, “married”, “loneliness”, and “social isolation”, and on later life engagement in routine health checks, immunisations, or cancer screening services with the terms “health check”, “immunis*”, “vaccin*”, “cancer screen*”, and “mammog*”. A systematic review and meta-analysis showed lower immunisation uptake among older people living alone than among those living with others. Several studies found higher participation in other preventive health services among married people than among unmarried people; however, few studies considered a wide range of social connectedness indicators including size of personal social networks or quality of social relationships. Cross-sectional studies were the most common study type.

**Added value of this study**

We examined participation in a range of preventive health-care programmes including routine health checks, immunisation, and cancer screening among older people (68–69 years) in the UK. This study included prospective measures of chronic exposure to low social connectedness and dynamics of social connectedness, which might be especially important in older age because employment and social roles typically change during this life stage.

**Implications of all the available evidence**

Older adults who are unmarried have a smaller personal social network, or have declining quality of social relationships, and engage less with preventive health services, including routine health checks, immunisations, and cancer screening programmes, than those with good social connectedness. National guidelines already recommend improved access to social networks for older people to maintain good mental health. This study supports public health initiatives to increase social networks because they could benefit individuals beyond mental health. Population segmentation based on indicators of social connectedness could help to improve targeting of initiatives to increase participation in preventive health care.

Different elements of social connectedness could be linked to uptake of different preventive health services according to the extent of invasiveness, consequences of a positive test result, cost, or mechanism for invitation to participate. We examined participation in a range of preventive health-care programmes including routine health checks, immunisation, and cancer screening among older people (68–69 years). In the UK, older people are encouraged to participate in several programmes because of the age-related increase in risk of chronic diseases. We aimed to examine cross-sectional and longitudinal associations between quantity and quality of social connectedness from ages 53 years to 69 years and engagement in preventive health services at 68–69 years.

## Methods

### Study design and participants

We used data from an observational, cohort study of 5362 socially stratified singleton births enrolled in March 1946 in England, Scotland, and Wales. One of the prespecified aims of the Medical Research Council National Survey of Health was to investigate the association of participation in preventive services with social connectedness. This Medical Research Council National Survey of Health and Development cohort was followed up prospectively 24 times from birth onwards, with the most recent follow-ups at ages 53 years, 60–64 years, and 68–69 years. At ages 68–69 years (in 2014–15), individuals reported participation in blood pressure and cholesterol measurement, eyesight and dental check-ups, influenza immunisation, and bowel and breast cancer screening. All measures used in this study were reported by participants. At 68–69 years, we obtained ethics approval from the National Research Ethics Service Queen Square REC (14/LO/1073) and Scotland A REC (14/SS/1009), and all participants provided written informed consent.

### Measures

#### Outcomes

Preventive health-care programmes differ across countries. We considered self-reported participation in seven preventive health services that were recommended by the National Health Service (NHS) in England, Scotland, and Wales for people aged 65–69 years at the time of data collection: blood pressure measurement and cholesterol measurement within the past 5 years, eyesight check-up within the past 2 years, dental check-up within the past year, immunisation against influenza within the past year, bowel cancer screening within the past 2 years, and women's breast cancer screening within the past 3 years. Except for dental check-ups, individuals did not have to pay for these services at the time of the study. A priori, we expected associations between social connectedness and participation in preventive health services to be broadly consistent across services. Our primary outcome measure therefore summed participation across all these tests and services at ages 68–69 years.

#### Exposures

Structural and functional aspects of social relationships were captured at ages 53 years, 60–64 years, and 68–69 years. At 68–69 years, we considered marital or cohabitation status, size of personal social network, frequency of face-to-face contact, parental status, and quality of social relationships. Size of social network was captured by the number of friends or relatives seen once a month or more with response options of zero, one to two, three to five, six to ten, and 11 or more. Reported frequency of face-to-face contact with relatives and friends (response options were never or almost never, once every few months, about once a month, about once a week, and almost daily, coded from 1 to 5) was summed and standardised. Quality of social relationships was based on three items from the Close Person's Questionnaire:^19^ whether the nominated closest person made them feel good about themselves, they had shared interests, or they had confided in the nominated person. Response options were coded from 0 (not at all) to 3 (a great deal), and then summed and standardised.

We derived cumulative exposure to small social network size by summing indicators for seeing fewer than three friends and relatives at ages 53 years, 60–64 years, and 68–69 years. Cumulative exposure to low social relationship quality was calculated by summing indicators for a value less than the mean at ages 53 years, 60–64 years, and 68–69 years.

#### Covariates

Socioeconomic disadvantage is associated with low engagement in preventive health care^7^ and poor social connectedness.^20^ We controlled for educational attainment captured at 26 years, household occupational social class at 53 years, because this mostly predates retirement-related transitions (based on the Registrar General's classification that groups occupations on the basis of their standing within the community from non-manual professional to manual unskilled occupations), and paid employment or non-employment at 68–69 years. Individuals previously identified as high risk or with a specific condition might have been recommended to attend particular checks more frequently than usual; therefore, doctor-diagnosed chronic conditions, including angina (reported from age 53 years onwards), high blood pressure (43 years onwards), and diabetes (36 years onwards), were adjusted for. We also controlled for number of consultations for a health problem with a general practitioner or another health-care professional at the general practice in the past 12 months. Women have more regular contact with health-care professionals for women's health and family health matters than do men for men's health and family health matters; therefore, we additionally controlled for sex. We also tested whether sex modifies any association between social connectedness and preventive health care as some evidence, albeit limited, suggests that the relevant components of connectedness differ for men and women.^21^

### Statistical analysis

The number of preventive health services attended is a count variable. We modelled the number not attended using Poisson regression (with 0 being the most common value) because it most closely follows the Poisson distribution. When exponentiated, Poisson regression estimates give the ratio of expected count per unit increase in exposure, referred to as the incident rate ratio (IRR). We first examined the association between each social connectedness indicator (one at a time) and non-participation in services (model 1). Interactions of sex by social connectedness were tested in each model. In model 2, we additionally controlled for potential confounders: education, household social class, employment status, doctor-diagnosed chronic illnesses, and number of general practitioner consultations for health problems in the past 12 months. In model 3, we also included all variables of social connectedness to identify those that showed the strongest associations with the outcome and might therefore be considered highest priority in future intervention studies. In sensitivity analysis, we excluded breast cancer screening to include services common to men and women. We also estimated the likelihood of non-participation in each service separately according to extent of social connectedness using logistic regression. For specific services, we excluded participants with prevalent blood pressure problems, high cholesterol concentrations, or any cancer.

Longitudinal data on social connectedness and non-participation in services were used in two ways. First, we tested whether increased cumulative exposure to low social connectedness is associated with increased risk of non-participation in services by including cumulative measures of small social networks or low quality of social relationships in models adjusted for sex, education, household socioeconomic position, employment status, doctor-diagnosed chronic illnesses, and number of general practitioner consultations for health problems in the past 12 months. In the second approach, we tested whether decline in social connectedness is related to increased risk of non-participation in services. We first generated estimates of the intercept and change in quality of social relationships between ages 53 years and 68–69 years using a random slopes model with linear age as a fixed and random parameter and an unstructured variance-covariance matrix for the random parameters. This method generated person-level random intercept (centred at 53 years) and slope estimates (indicating person-specific change in support), which were then standardised and used as independent variables in Poisson regression models with adjustment for the covariates.

2132 participants had complete data on participation in preventive health services and forms the main analytical sample for this study. Individuals lost to follow-up before ages 68–69 years had lower cognitive scores in childhood and adulthood, poorer physical health, and more health behavioural risk factors than did those included in the analysis; however, mental health symptoms between these groups did not differ.^22^ Individuals present at ages 68–69 years but with missing outcome data had lower educational attainment, less advantaged social class, and many or no general practitioner visits than did those included in the analysis; however, chronic conditions did not differ between these groups. Data on participation in cholesterol measurement were more frequently missing than for other services. Multiple imputation by chained equations was used to include participants with missing data on covariates and social connectedness on the basis of the assumption that the propensity for these data to be missing can be explained by the observed data (ie, data are missing at random). 20 datasets were generated and estimates combined by use of Rubin's rules. Outcome data were included in the imputation part of the model but not in the estimation part. STATA, version 14.0, was used for all analyses.

### Role of the funding source

The funders of the study had no role in study design, data collection, data analysis, data interpretation, or writing of the report. The corresponding author had full access to all the data in the study and had final responsibility for the decision to submit for publication.

## Results

By ages 68–69 years (in 2014–15), 1531 (29%) of 5362 participants had died or emigrated and therefore were not eligible to participate in the preventive health services of interest. 1699 (32%) of 5362 participants had withdrawn, were lost to follow-up, or did not complete outcome data (appendix p1). 2132 individuals reported participation in blood pressure and cholesterol measurement, eyesight and dental check-ups, influenza immunisation, and bowel and breast cancer screening. 940 (44%) of 2132 participants attended all preventive health services within the recommended timeframes (table 1). Influenza immunisation and bowel cancer screening were more commonly missed (564 [24%] of 2310 participants and 518 [23%] of 2302) than were the other services investigated. Prevalence of missed eyesight and dental checks was higher for men than for women, but the prevalence of missed cholesterol measurement was higher for women. Frequency of face-to-face social contact and social network size was lower in men than in women but the number of occasions with high social relationship quality was higher in men. Associations between social connectedness and the main covariates are summarised in the appendix (p 2).

**Table 1:** Medical Research Council National Survey of Health and Development participants with data on participation in preventive health services

		**All (n=2132)**	**Men (n=1022)**	**Women (n=1110)**	**p value**
Number of services not attended		..	..	..	p=0·50
	Zero (attended all services)	940/2132 (44%)	463/1022 (45%)	477/1110 (43%)	..
	One	627/2132 (29%)	288/1022 (28%)	338/1110 (31%)	..
	Two	290/2132 (14%)	142/1022 (14%)	147/1110 (13%)	..
	Three	164/2132 (8%)	81/1022 (8%)	82/1110 (7%)	..
	Four	68/2132 (3%)	29/1022 (3%)	40/1110 (4%)	..
	Five	30/2132 (1%)	12/1022 (1%)	17/1110 (2%)	..
	Six to seven	15/2132 (1%)	7/1022 (1%)	9/1110 (1%)	..
Service					
	Blood pressure measurement within past 5 years	148/2317 (6%)	67/1115 (6%)	81/1202 (7%)	p=0·47
	Cholesterol measurement within past 5 years	404/2268 (18%)	175/1088 (16%)	229/1180 (19%)	p=0·04
	Eyesight check-up within past 2 years	258/2306 (11%)	154/1097 (14%)	104/1209 (9%)	p=0·001
	Dental check-up within past year	346/2307 (15%)	190/1103 (17%)	157/1204 (13%)	p=0·01
	Influenza immunisation within past year	564/2310 (24%)	264/1097 (24%)	300/1213 (25%)	p=0·71
	Bowel cancer screen within past 2 years	518/2302 (23%)	253/1099 (23%)	265/1203 (22%)	p=0·57
	Mammogram within past 3 years	..	..	153/1201 (13%)	..
Marital or cohabitation status at 68–69 years		..	..	..	p<0·0001
	Not married or cohabiting	413/2101 (20%)	142/1011 (14%)	271/1090 (25%)	..
	Married or cohabiting	1688/2101 (80%)	869/1011 (86%)	819/1090 (74%)	..
	Missing	31/2132 (1%)	..	..	..
Parental status		..	..	..	p=0·06
	Does not have children	270/2102 (13%)	143/1002 (14%)	127/1100 (12%)	..
	Has child (ren)	1832/2102 (87%)	859/1002 (86%)	973/1100 (89%)	..
	Missing	30/2132 (1%)	..	..	..
Social network size at 68–69 years*		..	..	..	p=0·001
	Zero to two	401/2121 (19%)	219/1018 (22%)	182/1103 (17%)	..
	Three to five	694/2121 (33%)	349/1018 (34%)	345/1103 (31%)	..
	Six to ten	527/2121 (27%)	246/1018 (24%)	326/1103 (30%)	..
	11 or more	454/2121 (21%)	204/1018 (20%)	250/1103 (23%)	..
	Missing	11/2132 (1%)	..	..	..
Face-to-face contact					
	At 68–69 years	0·00 (1·00)	−0·10 (1·34)	0·11 (0·95)	p=0·04
	Missing	13/2132 (1%)	..	..	..
Cumulative number of occasions exposed to small network size at 53–69 years		..	..	..	p<0·0001
	Zero	1106/1662 (67%)	455/763 (60%)	651/899 (72%)	..
	One	363/1662 (22%)	193/763 (25%)	170/899 (19%)	..
	Two	129/1662 (8%)	81/763 (11%)	48/899 (5%)	..
	Three	64/1662 (4%)	34/763 (4·5%)	30/899 (3%)	..
	Missing ages 68–69 years	11/2132 (1%)	..	..	..
	Missing ages 60–64 years	391/2132 (18%)	..	..	..
	Missing ages 53 years	162/2132 (8%)	..	..	..
Social relationship quality at 68–69 years		0·00 (1·00)	0·08 (1·00)	−0·08 (0·99)	p<0·0001
Cumulative number of occasions exposed to low social relationship quality at 53–69 years		..	..	..	p<0·0001
	Zero	387/1588 (24%)	210/735 (29%)	177/833 (21%)	..
	One	338/1588 (21%)	163/735 (22%)	175/833 (21%)	..
	Two	359/1588 (23%)	166/735 (23%)	193/833 (23%)	..
	Three	504/1588 (32%)	196/735 (27%)	308/833 (37%)	..
	Missing ages 68–69 years	62/2132 (3%)	..	..	..
	Missing ages 60–64 years	374/2132 (18%)	..	..	..
	Missing age 53 years	229/2132 (11%)	..	..	..

Data are n/N (%) or mean (SD). Missing data do not total 100% because they were not included in the calculation of the distribution of each exposure for individuals with observed data.

* Number of relatives and friends seen once a month or more.

We found no evidence that associations between social connectedness at ages 68–69 years and participation in preventive health services differed between men and women (appendix p 3). The sex-adjusted IRR for non-participation in preventive health services for unmarried or not cohabiting participants compared with married or cohabiting participants was 1·33 (95% CI 1·20–1·47; table 2, model 1). Small networks (especially less than three friends and relatives) and not having children were also associated with increased non-participation. Frequent contact and high social relationship quality with the closest person were associated with low relative risk of non-participation. An IRR of 0·91 per 1 SD indicates that the 5% of individuals with scores 1·96 SDs less than the mean relationship quality missed 30% more preventive health service opportunities than did the 5% of those with scores 1·96 SDs more than the mean.

**Table 2:** Social connectedness and risk of non-participation in preventive health services at ages 68–69 years

		**Model 1**	**Model 2**	**Model 3**
Marital or cohabitation status				
	Married or cohabiting	1·00	1·00	1·00
	Not married or cohabiting	1·33 (1·20–1·47)*	1·28 (1·16–1·43)*	1·24 (1·11–1·37)*
Social network size†				
	Zero to two	1·51 (1·32–1·71)*	1·45 (1·27–1·65)*	1·31 (1·13–1·53)
	Three to five	1·16 (1·02–1·31)*	1·15 (1·02–1·31)*	1·11 (0·97–1·26)
	Six to ten	1·07 (0·94–1·21)	1·06 (0·93–1·21)	1·04 (0·91–1·19)
	11 or more	1·00	1·00	1·00
Any children				
	Yes	1·00	1·00	1·00
	No	1·23 (1·10–1·39)*	1·16 (1·09–1·23)*	1·12 (0·99–1·27)
Face-to-face contact (*Z* score)		0·92 (0·88–0·96)*	0·91 (0·87–0·95)*	0·98 (0·96–1·01)
Social relationship quality (*Z* score)		0·91 (0·87–0·95)*	0·95 (0·91–0·99)*	0·95 (0·91–0·99)*

Data are incident rate ratio (95% CI). Model 1 includes sex. Model 2 additionally includes education, occupational class, employment status, doctor-diagnosed chronic illnesses, and number of general practitioner consultations for health problems in the past 12 months. Model 3 additionally includes all social connectedness variables.

* p<0·05.

† Number of relatives and friends seen once a month or more.

High educational attainment was associated with low relative risk of non-participation (appendix p 4). Individuals with doctor-diagnosed conditions and those who had consulted a general practitioner one to five times or at least six times in the past 12 months had lower relative risk of non-participation than did individuals who had not consulted the doctor. However, adjustment for these covariates only partly attenuated the associations between social connectedness and participation (table 2, model 2). Estimates for social connectedness were further attenuated when all social connectedness variables were included (table 2, model 3). However, independent associations were seen between high relative risk of non-participation and being unmarried or not cohabiting, having a small social network size, and low quality of social relationships.

The direction of association between social connectedness and each preventive health service was mostly consistent in showing that likelihood of non-participation was high among individuals who were less socially connected, but most associations did not attain statistical significance (table 3). Estimates excluding those with blood pressure problems, high cholesterol, or cancer were similar (appendix p 5).

**Table 3:** Social connectedness and risk of non-participation in individual preventive health services at ages 68–69 years

		**Blood pressure measurement (n=2317)**	**Cholesterol measurement (n=2268)**	**Eyesight check-up (n=2306)**	**Dental check-up (n=2307)**	**Influenza immunisation (n=2310)**	**Bowel cancer screen (n=2302)**	**Mammogram (n=1201)**
Marital or cohabitation status								
	Married or cohabiting	1·00	1·00	1·00	1·00	1·00	1·00	1·00
	Not married or cohabiting	1·32 (0·88–2·00)	1·00 (0·89–1·12)	1·06 (0·92–1·22)	1·18 (1·05–1·32)	1·04 (0·95–1·16)	1·20 (1·09–1·32)	1·55 (1·05–2·28)
Social network size*								
	Zero to two	1·70 (0·99–2·91)	2·01 (1·39–2·89)	1·19 (0·78–1·80)	1·32 (0·93–1·88)	1·42 (1·04–1·94)	1·83 (1·33–2·52)	1·34 (0·77–2·32)
	Three to five	1·11 (0·64–1·93)	1·56 (1·12–2·16)	1·02 (0·70–1·48)	0·80 (0·57–1·12)	1·18 (0·89–1·55)	1·41 (1·05–1·90)	1·08 (0·66–1·77)
	Six to ten	1·70 (0·99–2·91)	1·16 (0·82–1·65)	1·00 (0·67–1·49)	0·77 (0·53–1·10)	1·02 (0·76–1·36)	1·23 (0·90–1·68)	0·85 (0·51–1·42)
	11 or more	1·00	1·00	1·00	1·00	1·00	1·00	1·00
Any children								
	Yes	1·00	1·00	1·00	1·00	1·00	1·00	1·00
	No	1·18 (0·72–1·96)	1·35 (0·98,1·85)	1·16 (0·79–1·69)	1·15 (0·81–1·64)	1·33 (1·01–1·75)	1·41 (1·06–1·87)	1·12 (0·65–1·94)
Face-to-face contact (*Z* score)		0·84 (0·71–0·99)	0·78 (0·69–0·87)	0·96 (0·84–1·09)	0·90 (0·80–1·00)	0·95 (0·86–1·05)	0·90 (0·81–0·99)	0·93 (0·78–1·11)
Social relationship quality (*Z* score)		0·93 (0·78–1·11)	0·96 (0·86–1·08)	0·85 (0·74–0·97)	0·81 (0·72–0·91)	0·91 (0·82–1·00)	0·86 (0·77–0·95)	0·90 (0·75–1·08)

Data are odds ratio (95% CI). Each social connectedness indicator is entered singly in model 2 adjusted for sex, education, occupational class, employment status, doctor-diagnosed chronic illnesses, and number of general practitioner consultations for health problems in the past 12 months.

* Number of relatives and friends seen once a month or more.

Association between number of occasions exposed to a small social network size at ages 53–69 years and risk of non-participation was graded, although 95% CIs for one, two, and three occasions overlapped (table 4). Cumulative exposure to low social relationship quality did not show a graded association with non-participation. Increasing quality of social relationships from ages 53 years to 69 years was associated with reduced risk of non-participation (IRR 0·93, 95% CI 0·89–0·97; table 4).

**Table 4:** Social connectedness assessed at ages 53–69 years and risk of non-participation in preventive health services at 68–69 years

	Incident rate ratio for non-participation (95% CI)*
**Number of occasions exposed to small network size at 53–69 years**†	
Zero	1·00
One	1·23 (1·06–1·42)‡
Two	1·36 (1·13–1·64)‡
Three	1·66 (1·30–2·11)‡
**Number of occasions exposed to low social relationship quality at 53–69 years**§	
Zero	1·00
One	1·19 (1·03–1·38)‡
Two	1·35 (1·17–1·55)‡
Three	1·25 (1·08–1·43)‡
**Social relationship quality (*Z* score)**	
Baseline (age 53 years)	0·93 (0·88–0·97)‡
Slope	0·93 (0·89–0·97)‡

Each connectedness indicator is entered in the model singly (not mutually adjusted); n=2132.

* Adjusted for sex, education, occupational class, employment status, doctor-diagnosed chronic illnesses, and number of general practitioner consultations for health problems in the past 12 months.

† Small network is none to two friends or relatives.

‡ p<0·05.

§ Low quality is less than the sample mean.

## Discussion

Social connectedness is associated with participation in preventive health services recommended for people older than 65 years. Controlling for socioeconomic factors and chronic disease, being unmarried or not cohabiting, having a small social network size, and having a low-quality relationship with the closest person were associated with non-participation in preventive health services. Changes in social relationship quality also affect participation in preventive health services; improvements in quality of social relationships were associated with increased participation.

This study benefits from longitudinal data, considered participation across many different preventive health programmes, and controlled for individual socioeconomic factors and chronic conditions. However, our study also has some limitations. Estimates from screening and immunisation programmes indicate that the sample population might have participated more in preventive health programmes than the general population. According to National General Practice Profile data, 91% of patients aged at least 45 years had a record of blood pressure measurement within the past 5 years. Approximately 50 000 NHS sight checks per 100 000 adults aged 60 years and older were completed in 2016–17; however, the recommendation for a sight check is every 2 years.^23^ Approximately 51% of adults were seen by an NHS dentist in the previous 2 years, but these data are not categorised into age groups and do not include private dental care.^24^ In 2016, in England, 58% and 76% of adults older than 65 years participated in screening for bowel cancer and breast cancer, respectively; uptake of influenza immunisation was similar to our study at 71%.^25^ The National Survey of Health and Development cohort might be more socially engaged and proactive in preserving their health than the general population; therefore, we might have underestimated associations between social connectedness and participation in preventive health care. Participation in some of these preventive health services follows an invitation from the service provider but we did not have data on invitations, nor could we control for rurality or other local variations in access to these preventive programmes. We controlled for socioeconomic factors that have been linked to access and uptake; however, residual confounding remains a possibility and the small effect sizes should be considered. Our measure of social network size was based on the number of relatives and friends seen and did not explicitly include coworkers or acquaintances. Although studies^26^ of cancer screening uptake suggest that these groups might not have a strong social influence, they could provide information and increase contact with people who have used the service or had the disease. Data on participation of social network members in specific screening programmes or other services and their experience of the disease were not available. Additionally, we were unable to include behaviours spread through online social media connectedness in our set of exposures. Studies suggest that information and influence through social media might increase and reduce vaccination uptake.^27^ Because of the small sample size, the study had low statistical power to examine participation in specific preventive services. Furthermore, prevalence of poor social connectedness was low in this cohort. Exposures and outcomes were self-reported, although we do not know how social desirability bias relates to social connectedness.

Using information on participation in routine health checks, immunisation, and screening programmes enabled us to show consistent patterns across different types of preventive health services. Improved social connectedness might promote participation in preventive health services through several pathways. In health behaviour models, behaviours such as participation in preventive health services are considered a result of individuals' intentions and abilities.^28^ Structural and functional elements of social connectedness plausibly contribute to a person's intention and ability to engage in preventive health services by influencing awareness, information, motivation, and action. Having a friend or relative who encouraged health check uptake is linked to greater intention to participate in cholesterol, blood pressure, and blood glucose checks in the next year.^15^ Spouses and family members influenced the decision to participate among attendees of colonoscopy screening,^29^, ^30^ and influence of family members might be particularly salient,^31^ possibly partly because of hereditary cancer risk. Absence of a partner, family, and important others who encourage cancer screening or who participate in cancer screening is associated with non-attendance or irregular attendance,^32^, ^33^ and protection of family members motivates influenza immunisation.^34^ Pathways linking social connectedness to a particular preventive health service might depend on the service of interest and particular barriers to participation in that service, including its acceptability (eg, how invasive or unpleasant a test or screening is, and concerns about the implications of the test result), the actual or perceived effectiveness of the service, other contextual barriers (eg, geographical access and stressful life events), and the nature of the social connection (eg, close or bonding social connections, or weak or bridging social connections). Exploration of these pathways was beyond the scope of the study; however, individuals who are not socially connected appear to be at greater risk of non-participation in many checks and screens than are individuals who are socially connected, which is in line with earlier studies.^8^, ^13^ Indicators of social connectedness, especially size of social networks, frequency of face-to-face contact, and parental status, were moderately correlated. Although these indicators were associated with participation in preventive health services in confounder-adjusted models, frequency of face-to-face contact and parental status were not associated independently of other social connectedness indicators. All three indicators plausibly promote awareness, motivation, and action; however, size of social networks emerged as the strongest correlate of participation in preventive health services in our analysis.

Our findings align with several previous studies showing greater likelihood of participation in routine health checks or cancer screening among married than unmarried people^12^ and add to the sparse literature on other aspects of the social network, including size of personal social networks. Small network size captured concurrently with participation in preventive health services was associated with reduced participation. The hypothesis that prolonged exposure to small social network size is associated with increased risk was not confirmed but warrants further investigation. Additionally, social connectedness can change as people age, and our analysis using repeat measures of quality of social relationship highlighted the association between declines in quality of social relationships and reduced participation in preventive health services.

National guidelines from the National Institute for Health and Care Excellence and Public Health England recommend that older people should have improved access to social contacts and networks and participate in social activities to maintain good mental health.^35^, ^36^ Positive social influence and social support are also being incorporated into initiatives to increase participation in screening and other preventive health programmes.^4^ Increasing social connectedness is also recognised as a cost-effective strategy to improve health.^37^ The estimated return on investment of £1·26 for every £1 spent on such programmes is very conservative because it takes into account only the potential health system costs of depression and self-harm associated with social isolation, yet individuals are also likely to have physical and cognitive health benefits.^37^ Our study suggests that improvement of access to social contacts and networks could additionally affect the health-care system by improving uptake of a range of evidence-based preventive health services. Structural and functional domains were independently associated with non-participation, suggesting that awareness, information, motivation, and emotional or practical support derived from social relationships might all be relevant through psychosocial pathways and practical support. Public health teams could therefore assess whether interventions to increase social contact (frequency and number) and improve the quality of existing social relationships that promote a sense of belonging can increase uptake of preventive health care. Providers of preventive health services might consider population segmentation based on social connectedness indicators to aid targeting of initiatives to increase participation. Individuals with poor social connectedness appear to be at greater risk of not engaging in the full range of preventive services than those with good social connectedness and thus might benefit from public health campaigns to increase awareness of and facilitate access to these services.

## Data sharing
