# Supplementary material for: Social connectedness and engagement in preventive health services: an analysis of data from a prospective cohort study
Source: Lancet Public Health. 2018 Aug 22;3(9):e438–46. doi: 10.1016/S2468-2667(18)30141-5 (PMC6123501; doi:10.1016/S2468-2667(18)30141-5)
Supplement: Supplementary appendix [file mmc1.pdf]

THE LANCET
Public Health

Supplementary appendix

This appendix formed part of the original submission and has been peer reviewed. We post it as supplied by the authors.

Supplement to: Stafford M, von Wagner C, Perman S, Taylor J, Kuh D, Sheringham J. Social connectedness and engagement in preventive health services: an analysis of data from a prospective cohort study. *Lancet Public Health* 2018; published online Aug 21. http://dx.doi.org/10.1016/S2468-2667(18)30141-5.

WEB EXTRA MATERIAL

Supplementary Figure 1. Flow chart

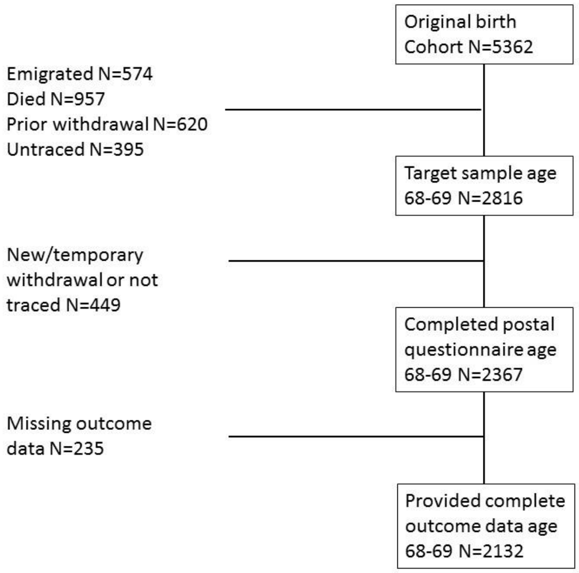

Supplementary Table A. Pairwise polychoric correlation coefficients

	2	3	4	5	6	7	8	9	10
1.Married	0.01	0.29	0.00	0.25	-0.26	0.08	0.10	-0.05	-0.06
2.Network size	1.00	0.16	-0.46	0.21	0.14	0.10	0.05	0.05	-0.03
3.Children		1.00	-0.38	0.02	-0.26	-0.08	0.03	0.03	0.01
4. Contact			1.00	-0.30	-0.07	-0.11	0.06	-0.03	0.02
5. Quality				1.00	-0.10	0.06	0.09	-0.05	-0.03
6.Female					1.00	-0.18	-0.07	-0.28	0.13
7.Education						1.00	0.55	0.12	-0.07
8.Social class							1.00	0.10	-0.01
9.Employed								1.00	-0.12
10. GP consultations									1.00

Network size, relationship quality, education, social class and GP consultations are coded low to high in this Table.

Supplementary Table B. Gender-stratified association between social connectedness and risk of non-participation in preventive health services at age 68-69. Each connectedness indicator is entered in model singly (not mutually adjusted)

	Incident rate ratio for non-participation (95% CI)[b]		P for gender x connectedness interaction
	Men (n=1022)	Women (n=1110)	
Marital status			0·54
Not married/cohabiting	1	1	
Married/cohabiting	**0.69 (0.59,0.82)**	**0.83 (0.73.0.94)**	
Social network size[a]			0·57
0-2	**1·44 (1·18,1·75)**	**1·46 (1.22,1.75)**	
3-5	**1·25 (1·04,1·50)**	1·05 (0·89,1.24)	
6-10	1·12 (0.92,1.37)	1·01 (0·85,1·20)	
11+	1	1	
Parental status			0.96
Has child(ren)	**0.81 (0.68,0.95)**	**0.79 (0.67,0.94)**	
Does not have children			
Face-to-face contact (z-score)	**0.91 (0.85,0.96)**	**0.91 (0.86,0.97)**	0.89
Social relationship quality (z-score)	**0·86 (0·81,0·92)**	0·95 (0·89,1·00)	0·07

[a]Number of relatives and friends seen once a month or more

[b]Adjusted for gender, education, occupational class, employment status, doctor diagnosed chronic illnesses, number of GP consultations for health problems in last 12 months; bold indicates p<0.05

Supplementary Table C. Socioeconomic characteristics, chronic disease, GP consultations for a health problem and risk of non-participation in preventive health services at age 68-69

	Incident rate ratio for non-participation (95% CI)
	Mutually adjusted
Men	1
Women	1·06 (0·97,1·16)
Educational attainment	
No formal qualifications	1
Ordinary secondary	**0·74 (0·66,0·83)**
Advanced secondary	**0·70 (0·62,0·79)**
Higher education	**0·69 (0·58,0·82)**
Occupational class	
Managerial/professional	1
Skilled non-manual	0·97 (0·82,1·15)
Skilled manual	**1·17 (1·03,1·33)**
Semi- and unskilled manual	1·13 (0·97,1·32)
Employment status	
Not in paid work	1
In paid work	**1.24 (1.12,1.37)**
Doctor diagnosed conditions	
Angina	**0·76 (0·62,0·95)**
Hypertension	**0·75 (0·68,0·82)**
Diabetes	**0·70 (0·60,0·82)**
Number of GP consultations in past 12 months	
0	1
1-5	**0·67 (0·61,0·73)**
6+	**0·59 (0·50,0·69)**

bold indicates $p<0.05$

Supplementary Table D. Social connectedness and risk of non-participation in individual preventive health services at age 68-69 excluding prevalent health condition

	Likelihood of non-participation			
	Blood pressure measurement; n=1282 OR (95% CI)	Cholesterol measurement n=1932 OR (95% CI)	Bowel cancer screen n=2041 OR (95% CI)	Mammogram n=1201 OR (95% CI)
Marital/cohabitation status				
Married	1	1	1	1
Not married	1.30 (082,2.04)	1.02 (0.90,1.15)	1.55 (1.21,2.00)	1.52 (1.02,2.27)
Social network size[a]				
0-2	1.92 (1.07,3.46)	1.98 (1.37,2.87)	1.83 (1.30,2.56)	1.41 (0.80,2.49)
3-5	1.13 (0.64,1.20)	1.59 (113,2.22)	1.38 (1.01,1.89)	1.10 (0.65,1.84)
6-10	1.43 (0.81,2.51)	1.10 (0.77,1.57)	1.23 (0.89,1.71)	0.92 (0.54,1.56)
11+	1	1	1	1
Any children				
Yes	1	1	1	1
No	1.23 (0.74,2.08)	1.32 (0.95,1.85)	1.41 (1.05,1.89)	1.02 (0.57,1.82)
Face-to-face contact (z-score)	0.84 (0.43,1.66)	0.78 (0.69,0.87)	0.88 (0.80,0.98)	0.93 (0.77,1.11)
Social relationship quality (z-score)	0·89 (0·74,1·07)	0·95 (0·84,1·07)	0·85 (0·77,0·95)	0.92 (0.76,1.11)

[a]Number of relatives and friends seen once a month or more

Each social connectedness indicator is entered in model singly, adjusted for gender, education, occupational class, employment status, doctor diagnosed chronic illnesses, number of GP consultations for health problems in last 12 months
